# A Single Point Mutation in GraS Drives Co-Evolution of Vancomycin Resistance and Virulence in *Staphylococcus aureus*

**DOI:** 10.3390/microorganisms14051151

**Published:** 2026-05-19

**Authors:** Zhen Hu, Yifan Rao, Lu Liu, Zuwen Guo, Yuting Wang, Weilong Shang, Huagang Peng

**Affiliations:** 1 Key Laboratory of Microbial Engineering Under the Educational Committee in Chongqing, Department of Microbiology, College of Basic Medical Sciences, Army Medical University (Third Military Medical University), Chongqing 400038, China; huzhenzhen1314@tmmu.edu.cn (Z.H.);; 2 Department of Emergency Medicine, The Second Affiliated Hospital, Army Medical University, Chongqing 400037, China; raoyifan1995@tmmu.edu.cn

**Keywords:** vancomycin-intermediate *Staphylococcus aureus*, GraSR, vancomycin resistance, virulence

## Abstract

The emergence of vancomycin-intermediate *Staphylococcus aureus* (VISA) threatens the efficacy of this last-line antibiotic. The GraSR two-component system is frequently mutated in VISA strains. Here, we demonstrate that the GraS(T136I) point mutation, identified in the clinical VISA isolate XN108, is a key determinant of reduced vancomycin susceptibility. Introducing this mutation into the susceptible strain Newman increased the vancomycin MIC from 1.5 to 4 mg/L, while its reversion in XN108 decreased the MIC from 12 to 8 mg/L. The mutation conferred common phenotypes, including thickened cell wall, decreased autolysis, and reduced cell surface negative charge via upregulation of the *dltABCD* operon and *mprF*. Notably, the GraS(T136I) mutation also upregulated virulence genes (*efb*, *hlb*, *sbi*, *hld*) and enhanced hemolytic activity. Interestingly, despite this hypervirulent profile, the mutant showed impaired long-term survival within macrophages. Our study reveals that a single GraSR mutation can co-regulate vancomycin resistance and virulence, offering new insights into the adaptation of *S. aureus* to antibiotic pressure.

## 1. Introduction

*Staphylococcus aureus* causes a broad spectrum of infections, ranging from mild cutaneous and soft tissue manifestations to life-threatening conditions, including bacteremia, endocarditis, sepsis, and toxic shock syndrome [1,2]. As methicillin-resistant *S. aureus* (MRSA) has emerged and spread globally, vancomycin has become a critical last-line therapy for severe *staphylococcal* infections [3,4]. In recent decades, however, the rising prevalence of vancomycin-resistant *S. aureus* (VRSA), vancomycin-intermediate *S. aureus* (VISA) and heterogeneous VISA (hVISA) strains poses a serious clinical challenge, often correlating with treatment failure and poor patient outcomes [5]. Thus, understanding the bacterial mechanisms underlying vancomycin resistance is essential for developing novel strategies to combat these life-threatening infections.

The first VRSA isolate in the United States was reported in 2002; since then, a total of 52 VRSA strains carrying van genes have been reported [6]. The molecular mechanism mediating vancomycin resistance in VRSA has been clearly elucidated. VRSA is maintained by retaining an original *enterococcal* plasmid or by transposition of Tn1546 from the VRE plasmid into a *staphylococcal* resident plasmid [7]. This plasmid carries vancomycin resistance genes, including the *vanA* operon, which encodes enzymes responsible for modifying or eliminating the vancomycin-binding site. Since the first documentation of VISA in 1997, numerous studies have identified the molecular drivers of reduced vancomycin susceptibility [8]. VISA strains typically display conserved phenotypic traits, such as thickened cell walls, diminished autolysis, altered cell surface charge, and attenuated virulence [9,10,11]. Genomic comparisons between vancomycin-susceptible *S. aureus* (VSSA)/VISA isolates have revealed recurrent mutations in regulatory genes [12,13], especially those encoding two-component systems (TCSs) such as *vraSR*, *graSR*, and *walKR* [14,15,16]. Additional mutations have been identified in genes involved in cell wall metabolism (*sle1*), central metabolism (*cmk*, *fdh2*), and transcriptional regulation (*rpoB*) [17,18,19].

TCSs are key bacterial signaling pathways that enable adaptation to environmental stresses, including antibiotic exposure [20]. In *S. aureus*, GraSR TCS plays a central role in modulating both antimicrobial resistance and virulence [21,22]. GraSR activates the *dltABCD* operon, which mediates D-alanylation of wall teichoic acids (WTAs) and lipoteichoic acids (LTAs), and the *mprF* gene, which catalyzes lysinylation of membrane phospholipids [23,24]. These modifications enhance the positive charge on the bacterial surface, reducing the binding of cationic antimicrobial peptides (CAMPs) and contributing to CAMP resistance. Clinically, mutations in *graSR* have been repeatedly identified in VISA strains. For instance, the GraR(N197S) substitution was shown to convert an hVISA strain into a fully VISA phenotype, highlighting its direct role in resistance development [25]. Beyond resistance, GraSR also governs the expression of multiple virulence determinants, including α-hemolysin, enterotoxins, and adhesins, thereby influencing host colonization, immune evasion, and disease progression [22].

While GraSR mutations are increasingly recognized in VISA, the functional consequences of most allelic variants remain poorly characterized. Specifically, the GraS(T136I) substitution is localized within the histidine kinase domain of GraS, which is an indispensable region for signal transduction and TCS activation [26]. The GraS(T136I) variant has been identified in clinical VISA isolates, yet its functional contribution to vancomycin resistance and virulence regulation remains experimentally unvalidated. Previous studies have focused on GraR mutations or other GraS variants, leaving the impact of GraS(T136I) on VISA phenotypic traits and pathogenicity unresolved [25,27]. This knowledge gap hinders a comprehensive understanding of how GraSR evolves to coordinate resistance and virulence in *S. aureus*, a critical step for targeting this pathway therapeutically.

In a previous study, we characterized the ST239 SCCmec-III VISA strain XN108, which exhibits a vancomycin MIC of 12 mg/L [28]. Genome sequencing identified several non-synonymous mutations, including WalK(S221P), GraS(T136I), and RpoB(H481N), that were hypothesized to contribute to its VISA phenotype [29]. In this study, we aimed to explore the functional role of GraS(T136I) in the VISA strain XN108. Using allelic exchange, we introduced this mutation into the VSSA strain Newman and reversed it in XN108. We demonstrate that GraS(T136I) is sufficient to confer reduced vancomycin susceptibility, accompanied by hallmark VISA phenotypes: thickened cell wall, decreased autolysis, and reduced cell surface negative charge. Moreover, this mutation upregulates key virulence genes and enhances hemolytic activity, indicating a dual role in antibiotic resistance and pathogenicity. Our data establish GraS(T136I) as a critical determinant linking vancomycin resistance and virulence in *S. aureus*.

## 2. Materials and Methods

### 2.1. Bacterial Strains and Growth Conditions

The VISA strain XN108 was isolated from wound secretion samples of a burn patient with severe *S. aureus* infection on 23 February 2004, and identified through a laboratory-based retrospective study approved by the Ethics Committee of the First Affiliated Hospital of Army Medical University (protocol (A)KY2024043) [28]. This whole-genome sequence of *S. aureus* XN108 is deposited in NCBI GenBank under the accession no. CP007447 [30]. The VSSA strain Newman was provided by Prof. Lu Yu (Jilin University). We used the restriction modification-deficient *S. aureus* strain RN4220 as an intermediate host for plasmid construction. *Escherichia coli* DH5α was used for general cloning. The *E. coli–S. aureus* shuttle vector pBT2 served as the allelic replacement vector. *S. aureus* strains were routinely cultured in brain heart infusion (BHI, Oxoid, Hampshire, UK) at 37 °C or 30 °C with shaking. When required, chloramphenicol (20 μg/mL) was added. *E. coli* strains were grown in Luria–Bertani (LB) medium supplemented with ampicillin (100 μg/mL) as needed. All experiments were performed in a BSL-2 laboratory in accordance with institutional biosafety regulations.

### 2.2. Genetic Manipulation of S. aureus

The GraS(T136I) mutation was introduced into the VSSA strain Newman using a previously described allelic replacement method [29]. Briefly, the *graS* gene carrying the T136I mutation was amplified by PCR from XN108 genomic DNA using primers pBT2-GraS-5 (CCGGAATTCACTAAATGATATTGGGTGATATGG) and pBT2-GraS-3 (GCGGGATCCGTATATCAGATAATTCCTTGTTTG). The PCR product and pBT2 were digested with EcoRI and BamHI, then ligated using T4 DNA ligase and cloned into the temperature-sensitive *E. coli–S. aureus* shuttle vector pBT2. The resulting plasmid, pBT2-GraS(T136I), was electroporated into *S. aureus* RN4220 under the conditions of 2.5 kV, 200 Ω, and 25 µF. Immediately after electroporation, 1 mL of BHI medium was added to the electroporated cells for a 1 h recovery at 30 °C. The recovered cells were then spread onto BHI agar plates supplemented with 20 µg/mL chloramphenicol and incubated at 30 °C for 24 h to allow single colony formation. Individual colonies were picked and inoculated into 2 mL of BHI liquid medium containing 20 µg/mL chloramphenicol, followed by incubation at 30 °C with shaking at 200 rpm for 18 h. Plasmids were extracted and purified, and subsequently electroporated into Newman under the conditions of 2.5 kV, 200 Ω, and 50 µF. After transformation of pBT2-GraS(T136I) plasmid into *S. aureus* Newman, the integration of the plasmid into the bacterial chromosome was induced by cultivating plasmid-carried Newman at 42 °C for 20 h, followed by cultivation at 25 °C for 20 h to generate the mutant strain Newman-GraS(T136I). The reciprocal revertant XN108-GraS(I136T) was constructed in a similar manner, using the wild-type *graS* allele from Newman. All constructs were verified by PCR amplification and Sanger sequencing.

### 2.3. Antibiotic Susceptibility Assay

Vancomycin MICs were determined by E-test on BHI agar plates according to Clinical and Laboratory Standards Institute (CLSI) guidelines. Fresh bacterial suspensions were adjusted to approximately 1 × 10^6^ CFU/mL, and 100 μL was spread evenly onto BHI agar. A vancomycin E-test strip (Liofilchem, Roseto degli Abruzzi, Italy) was placed in the center of the inoculated plate. Plates were incubated at 37 °C for 24 h, and the MIC value was read at the point where the inhibition ellipse intersected the strip.

### 2.4. Triton X-100 Stimulated Autolysis Assay

Autolysis was performed as previously described with some modifications [31]. Strains were first grown overnight in BHI liquid medium at 37 °C with shaking at 200 rpm. The overnight cultures were diluted 1:100 into fresh BHI medium and incubated at 37 °C with shaking at 200 rpm until an optical density was reached at 600 nm (OD_600_) of 1.0. The cells were collected by centrifugation at 14,000× *g* for 1 min. The supernatant was discarded, and the pellet was washed twice with 0.05 M Tris–HCl buffer (pH 7.5). The washed pellet was then resuspended in an equal volume of 0.05 M Tris–HCl buffer (pH 7.5) supplemented with 0.05% (*w*/*v*) Triton X-100 (Sigma-Aldrich, St. Louis, MO, USA) and incubated at 37 °C with constant shaking at 200 rpm. The decrease in the optical density at 600 nm (OD600) was measured each hour using a microplate reader (SpectraMax^®^M2/M2e, Sunnyvale, CA, USA). The experiment was repeated at least three times with similar results.

### 2.5. Transmission Electron Microscopy (TEM)

*S. aureus* cells were grown in BHI to the exponential growth phase, harvested, and fixed with 2.5% glutaraldehyde in 0.1 M phosphate buffer (pH 7.2) overnight at 4 °C, as previously described [32]. Samples were washed, post-fixed with 1% osmium tetroxide, dehydrated in a graded ethanol series, and embedded in epoxy resin. Ultrathin sections (approximately 70 nm) were cut and stained with uranyl acetate and lead citrate. The bacterial cell wall thickness was observed by transmission electron microscopy (JEOL, Tokyo, Japan) and measured using ImageJ software (version 1.54r).

### 2.6. Cytochrome C Binding Assay

Cell surface charge was assessed via the cytochrome C binding assay as previously described [33]. Briefly, exponential-phase bacteria were washed twice with 20 mM 3-(N-morpholino) propanesulfonic acid (MOPS) buffer (pH 7.0) and adjusted to an OD600 of 14 in the same buffer. Cytochrome C (Macklin, China) was added to a final concentration of 0.5 mg/mL. After 10 min incubation at room temperature, samples were centrifuged at 18,000× *g* for 6 min, and the absorbance of the supernatant was measured at 530 nm. A control without bacteria was used to determine 100% unbound cytochrome C. The percentage of bound cytochrome C was calculated from three independent experiments, each performed in triplicate.

### 2.7. RNA Extraction and Rt-Qpcr Determination

Total RNA was extracted from exponential-phase bacterial cultures using the SV Total RNA Isolation System (Promega, Madison, WI, USA). Cells were lysed in 0.5 mL nuclease-free water with 0.1 mm silica beads in a FastPrep 24 homogenizer (MP Biomedicals, Irvine, CA, USA). RNA (500 ng) was reverse-transcribed with random primers using a RevertAid First Strand cDNA Synthesis Kit (Thermo Fisher Scientific, Waltham, MA, USA). Quantitative PCR (qPCR) was performed with SsoAdvanced^TM^ Universal SYBR Green Supermix (Bio-Rad, Hercules, CA, USA) on a CFX96^TM^ Real-Time PCR Detection System (Bio-Rad, Hercules, CA, USA). Melting curve analysis was performed after qPCR amplification. The system was incubated at 65 °C for 5 s, subsequently heated to 95 °C and incubated for 5 min. Fluorescence signals were monitored throughout the procedure, and the specificity of amplification was determined by the melting curve peak profile. The primers used are listed in Table 1. The housekeeping gene *pta* was used for normalization. We repeated the assays using three independent biological samples. Relative gene expression levels were calculated using the 2^−ΔΔCt^. The relative expression was normalized to the reference gene and presented as a fold change relative to the control group. Statistical analyses were performed based on Student’s *t*-test to determine the significance of the gene expression levels, where *p* < 0.05 was considered statistically significant.

### 2.8. Hemolytic Activity

Hemolytic activity was performed as previously described [34]. Overnight cultures of *S. aureus* XN108 and its derivatives were diluted 1: 100 in fresh BHI and grown at 37 °C for 16 h. Supernatants were obtained by centrifugation (14,000× *g*, 1 min). A 100 μL aliquot of each supernatant was mixed with 900 μL of PBS containing 3% (*v*/*v*) sheep red blood cells and incubated at 37 °C for 20 min. PBS alone and ddH_2_O containing 3% sheep red blood cells served as negative and positive controls, respectively. After centrifugation (5000× *g*, 4 °C, 10 min), the OD543 value of the supernatant was measured. Hemolysis percentage was calculated as [(ODsample − ODnegative control)/(ODpositive control − ODnegative control)] × 100.

### 2.9. Macrophage Infection Assay

The macrophage infection assay was performed as previously described [35]. Briefly, RAW264.7 macrophages were cultured in high-glucose Dulbecco’s modified Eagle’s medium (DMEM, Thermo Scientific, Hercules, USA) supplemented with 10% (*v*/*v*) fetal bovine serum at 37 °C with 5% CO_2_. For infection, exponentially growing bacteria were washed twice with PBS and resuspended in PBS. RAW264.7 cells were infected with *S. aureus* at a multiplicity of infection (MOI) of 10 in 24-well plates. After 4 h of incubation at 37 °C with 5% CO_2_, the supernatant was removed, and the cells were washed with PBS. Fresh DMEM containing lysostaphin (1 mg/mL) and gentamicin (50 μg/mL) was added to eliminate extracellular *S. aureus*. Macrophages were then cultured for 4 or 12 h at 37 °C with 5% CO_2_. After lysis with PBS supplemented with 1% (*v*/*v*) Triton X-100 (Sigma-Aldrich, St. Louis, MI, USA), serial dilutions of the lysates were plated onto BHI agar plates. Bacterial colony-forming units (CFUs) were enumerated following incubation at 37 °C for 24 h, and the experiment was performed in triplicate.

### 2.10. Statistical Analysis

All experiments were repeated at least three times, and the data are expressed as mean ± standard deviation (SD). Student’s *t*-test was used for statistical comparison between two independent groups, and all quantitative data were analyzed using GraphPad Prism 9.0. A *p* value of less than 0.05 was considered statistically significant.

## 3. Results

### 3.1. Gras(T136I) Mutation Confers Reduced Vancomycin Susceptibility in S. aureus

**Figure 1:**
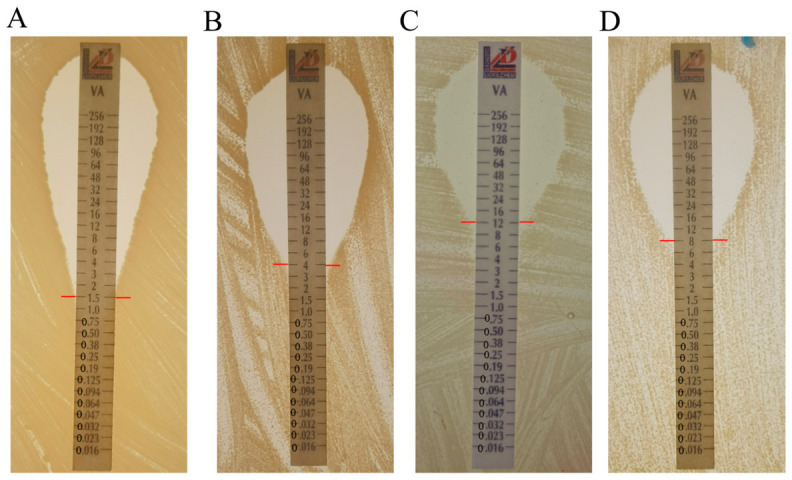
GraS(T136I) mutation reduces vancomycin susceptibility in *S. aureus*. Vancomycin susceptibilities of *S. aureus* strain Newman (**A**), Newman-GraS(T136I) (**B**), XN108 (**C**) and XN108-GraS(I136T) (**D**) were determined by E-test. Strains were grown overnight in BHI medium prior to MIC determination.

We previously identified that WalK(S221P), GraS(T136I), and RpoB(H481N) were speculated to contribute to the VISA phenotype of XN108. To assess the impact of the GraS(T136I) point mutation on vancomycin resistance, we introduced plasmids harboring this mutation into the VSSA strain Newman to generate the mutant strain Newman-GraS(T136I) through homologous recombination. Compared to the parental Newman strain (vancomycin MIC = 1.5 mg/L), Newman-GraS(T136I) exhibited increased vancomycin resistance, with an MIC of 4 mg/L (Figure 1A,B). Conversely, we introduced the wild-type GraS(I136T) allele from the VSSA strain Newman into XN108, generating the revertant strain XN108-GraS(I136T). This revertant displayed a reduced vancomycin MIC of 8 mg/L, compared to 12 mg/L for the parental XN108 strain (Figure 1C,D). These complementary genetic experiments confirm that the GraS(T136I) single-point mutation is directly responsible for the observed alterations in vancomycin MIC.

### 3.2. Impact of Gras(T136I) Mutation on Typical Visa Phenotypes

**Figure 2:**
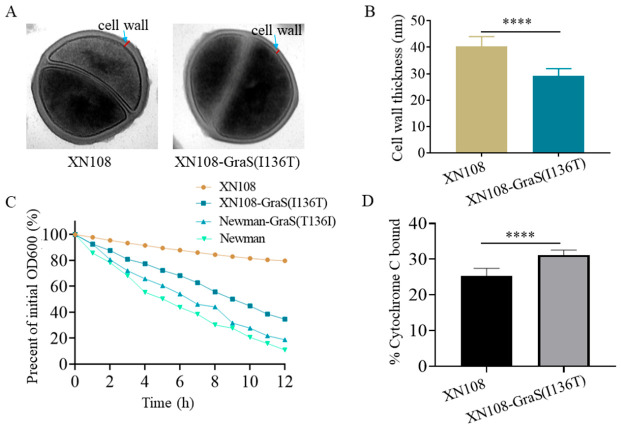
Effects of the GraS(T136I) mutation on typical VISA phenotypes. (**A**) TEM images of XN108 and the reverse mutant XN108-GraS(I136T). (**B**) Comparison of cell wall thickness between XN108 and XN108-GraS(I136T) (*n* = 15 for each strain). (**C**) Autolysis assay stimulated by Triton X-100. The autolysis rate was measured in XN108, XN108-GraS(I136T) Newman, and Newman-GraS(T136I), as described in the Section 2. Results are from three independent experiments, each performed in triplicate. (**D**) Surface charge assessed by cytochrome C binding assay. Data are presented as mean ± SD. Statistical significance was determined using a two-tailed Student’s *t*-test, with XN108 as the reference. **** *p* < 0.0001.

VISA strains exhibit common phenotypic traits, such as thickened cell walls and reduced autolytic activities [12]. To investigate whether GraS(T136I) confers these phenotypes, we performed TEM on exponentially growing cells. The cell walls of the revertant strain XN108-GraS(I136T) were significantly thinner than those of the parental XN108 strain (Figure 2A,B). Quantitative measurements confirmed this observation, with mean cell wall thicknesses of 40.28 ± 3.78 nm for XN108 and 29.08 ± 2.72 nm for XN108-GraS(I136T). Given that cell wall thickening is generally accompanied by decreased autolysis, we assessed the autolytic activity using the Triton X-100-induced autolysis assay. The autolysis rate of XN108 was significantly lower than that of the XN108-GraS(I136T) revertant (Figure 2C). Accordingly, the autolytic activity of Newman-GraS(T136I) was also decreased compared with the parental Newman strain (Figure 2C).

Beyond cell wall remodeling, alterations in cell surface charge represent another key mechanism of vancomycin resistance. Vancomycin, a positively charged glycopeptide, binds less efficiently to bacterial surfaces with increased positive charge. To verify this mechanism, we detected relative cell surface charge using a cytochrome C binding assay. Strain XN108 bound significantly less cytochrome C than the XN108-GraS(I136T) revertant (Figure 2D), indicating that XN108 possesses a less negatively charged cell surface. This result supports the model where GraS(T136I)-mediated reduction in surface negative charge contributes to decreased vancomycin affinity and enhanced resistance.

### 3.3. Gras(T136I) Alters Expression of Genes Controlling Cell Surface Charge and Virulence

**Figure 3:**
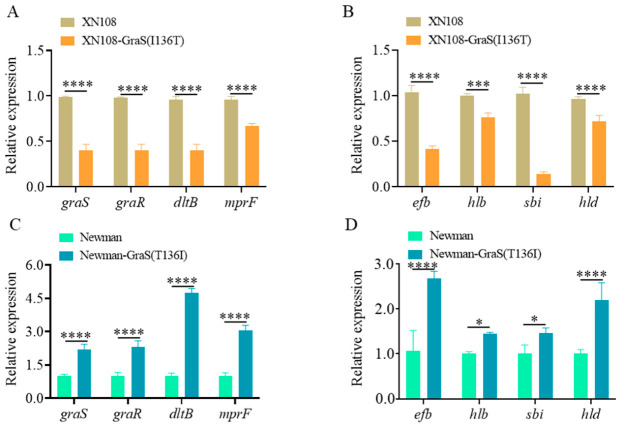
GraS(T136I) influences the expression of genes associated with cell surface charge and virulence. (**A**) Transcript levels of *graS*, *graR*, the *dlt* operon, and *mprF* in XN108 and XN108-GraS(I136T) during the exponential phase, as determined by RT-qPCR. (**B**) Transcript levels of virulence-related genes in XN108 and XN108-GraS(I136T). (**C**) Transcript levels of *graS*, *graR*, the *dlt* operon, and *mprF* in Neman and Newman-GraS(T136I). (**D**) Transcript levels of virulence-related genes in Neman and Newman-GraS(T136I). Data are presented as mean ± SD. Statistical significance was analyzed with a two-tailed Student’s *t*-test, using XN108 as the reference. * *p* < 0.05, *** *p* < 0.001, **** *p* < 0.0001.

The GraSR two-component system is known to be upregulated in VISA isolates [36]. Activation of GraSR enhances vancomycin resistance primarily by directly binding to and upregulating the *mprF* and *dltABCD* operon, which are involved in modulating cell surface charge modification and peptidoglycan metabolism [33]. To investigate whether the GraS(T136I) mutation affects the expression of these genes, we quantified relative transcript levels in XN108, XN108-GraS(I136T), Newman-GraS(T136I), and Newman using RT–qPCR. The transcript levels of *graS*, *graR*, *dltB* and *mprF* were significantly higher in XN108 compared to the revertant strain (Figure 3A). Similarly, the expression levels of these genes were also significantly higher in Newman-GraS(T136I) than in the parental Newman strain (Figure 3C). This result is consistent with the findings of the cytochrome C binding assay, confirming that the GraS(T136I) mutation activates the GraSR system, leading to the upregulation of *dltABCD* and *mprF*. This transcriptional reprogramming subsequently modulates cell surface charge and culminates in increased vancomycin resistance.

The GraSR system also modulates *S. aureus* pathogenesis [22]. Previous reports indicate that virulence-related genes, such as *efb*, *hlb*, *sbi*, and *hld*, are under GraSR regulation, as their expression was downregulated in a *graSR* deletion mutant. To examine the impact of the GraS(T136I) mutation on virulence, we determined the transcript levels of these genes. The results indicated that all tested virulence genes were significantly upregulated in XN108 relative to the XN108-GraS(I136T) revertant (Figure 3B). Likewise, these genes were also significantly upregulated in Newman-GraS(T136I) compared with the parental Newman strain (Figure 3D). This result indicates that the GraS(T136I) point mutation promotes vancomycin resistance and upregulates the expression of key virulence determinants.

### 3.4. Gras(T136I) Enhances Hemolytic Activity and Impairs Intracellular Survival in Macrophages

**Figure 4:**
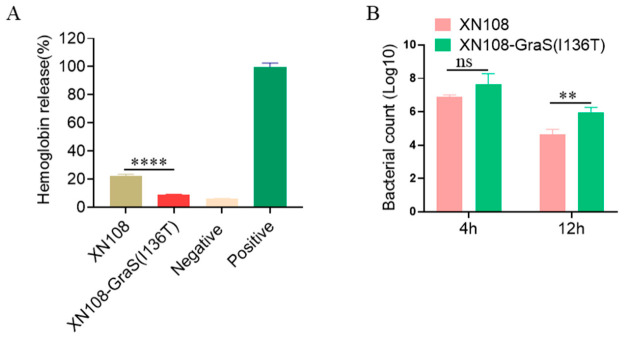
GraS(T136I) mutation enhances *S. aureus* virulence and impairs host cell invasion. (**A**) Hemolytic activity of XN108 and XN108-GraS(I136T) strains determined after 1 h incubation with 3% sheep red blood cells. PBS- and ddH_2_O-treated sheep red blood cells served as negative and positive controls, respectively. (**B**). Bacterial counts of XN108 and XN108-GraS(I136T) strains 4 and 12 h post phagocytosis by RAW264.7 macrophages. Data represent the mean ± SD (*n* = 3). Statistical significance was calculated by two-way ANOVA. ** *p* < 0.01, **** *p* < 0.001, and ns indicates no significance.

Given the upregulation of hemolysin genes in XN108, we compared the hemolytic activities of XN108 and XN108-GraS(I136T). As shown in Figure 4A, the XN108 strain exhibited significantly higher hemolytic activity compared to the XN108-GraS(I136T) revertant. Macrophages serve as a primary defense against bacterial invasion. To determine whether GraS(T136I)-mediated upregulation of virulence genes affects bacterial survival within host cells, we assessed the intracellular survival of both XN108 and XN108-GraS(I136T) strains within RAW264.7 macrophages. After 4 h of co-culture, the survival rates of XN108 and XN108-GraS(I136T) were comparable. However, after 12 h of culture, the intracellular survival of XN108 was reduced compared to the XN108-GraS(I136T) revertant (Figure 4B). Although the GraS(T136I) mutation enhances virulence factor expression and hemolytic activity, it unexpectedly reduces long-term survival of *S. aureus* within macrophages, potentially impairing sustained cellular invasion.

## 4. Discussion

*S. aureus* is a remarkably adaptable pathogen capable of evolving resistance to last-line antibiotics while maintaining or even enhancing its virulence potential [37,38,39,40,41]. Since the 1980s, the widespread use of vancomycin has driven the emergence of VISA and hVISA strains, posing a major global public health threat [18,42]. Although comparative genomics has identified numerous mutations associated with the VISA phenotype, only a subset has been functionally investigated. Among these, mutations in the GraSR regulatory system have emerged as a recurrent theme [8]. The GraR(N197S) substitution in the clinical VISA strain Mu50, for example, was shown to convert the hVISA strain Mu3 into a VISA phenotype upon genetic complementation [25]. However, how specific *graSR* mutations affect vancomycin resistance and other bacterial traits remains unclear.

In this study, we show that the GraS(T136I) point mutation, which was identified in the clinical VISA isolate XN108, is sufficient to elevate vancomycin resistance in the otherwise susceptible strain Newman, converting it to a VISA phenotype. Conversely, reverting this mutation in XN108 to the wild-type GraS(I136T) significantly reduced resistance (Figure 1). Notably, the vancomycin MIC of Newman-GraS(T136I) remained lower than that of parental XN108. This finding underscores the polygenic nature of VISA development, wherein multiple mutations collectively fine-tune cell wall homeostasis and surface properties to reduce drug access [29].

The GraSR is a TCS associated with glycopeptide resistance [33]. Primarily, GraSR enhances cell envelope charge through transcriptional control of the *dltABCD* operon and *mprF* [33]. These genes promote the incorporation of D-alanine into teichoic acids and lysine into membrane lipids, respectively, thereby increasing the positive charge of the cell surface [23,24]. Consistent with earlier reports [33,36], we found that GraS(T136I) activates the GraSR regulon, upregulating *graS*, *graR*, *dltB* and *mprF* (Figure 3A). Correspondingly, the XN108 strain exhibited a less negatively charged surface than the GraS(I136T) revertant, as measured by cytochrome C binding (Figure 2D). Since vancomycin is a positively charged glycopeptide, this charge repulsion likely contributes to reduced drug binding and higher MICs. Moreover, GraS(T136I) promoted cell wall thickening and diminished autolytic activity (Figure 2A–C), two characteristic features of VISA that may further restrict vancomycin access to its target.

Notably, the GraS(T136I) mutation resides within the histidine kinase domain of GraS, which is a conserved region responsible for ATP binding, autophosphorylation, and downstream signal transduction to GraR [28,43]. Histidine kinase activity is tightly regulated by conformational changes induced by environmental signals, and substitutions within this domain can alter TCS activation kinetics or ligand sensitivity [20,21]. Our transcriptional data showing upregulated *graSR* expression suggest that GraS(T136I) may enhance GraSR pathway activation, either by increasing autophosphorylation efficiency or reducing feedback inhibition. This gain-of-function mechanism aligns with previous observations of GraR(N197S)-mediated VISA development and highlights that diverse GraSR mutations can converge on pathway hyperactivation to promote vancomycin resistance.

Beyond its role in resistance, the GraSR system also modulates *S. aureus* virulence, though the phenotypic consequences of specific *graS* mutations have been less thoroughly explored [22]. Here, we show that GraS(T136I) significantly upregulates the expression of virulence-associated genes (*efb*, *hlb*, *sbi*, *hld*) and enhances hemolytic activity (Figure 3B and Figure 4A). This suggests that the mutation not only aids bacterial survival under antibiotic pressure but may also augment pathogenic potential. Interestingly, however, the enhanced virulence profile did not translate into improved intracellular survival in macrophages. Instead, XN108 showed markedly reduced persistence in RAW264.7 cells compared to the GraS(I136T) revertant after 12 h of infection (Figure 4B). These findings imply that while GraS(T136I) upregulates certain toxin genes, it may simultaneously impair adaptations required for long-term intracellular niche establishment, a trade-off that warrants further investigation.

## 5. Conclusions

In summary, this study demonstrates how a single nucleotide substitution in the GraS sensor kinase can simultaneously alter vancomycin susceptibility and virulence in *S. aureus*. The GraS(T136I) mutation is sufficient to confer a VISA phenotype, characterized by thickened cell wall, reduced autolysis, and decreased net negative surface charge, changes mediated through GraSR-dependent upregulation of *dltABCD* and *mprF*. Additionally, the same mutation enhances the expression of key virulence genes and increases hemolytic activity, indicating a coordinated remodeling of both resistance and pathogenicity programs. These findings illustrate the multifunctional role of GraSR in *S. aureus* adaptation and highlight how point mutations in global regulators can have pleiotropic effects on bacterial fitness, resistance, and virulence, which has important potential clinical value for the management of VISA infections. The GraS(T136I) mutation may serve as a target for screening potent inhibitors to develop adjuvant therapeutic strategies that restore vancomycin efficacy and attenuate bacterial virulence. Unraveling such connections may inform new strategies for countering the concurrent evolution of antibiotic resistance and virulence in this formidable pathogen.

Although this study clarified the role of the GraS(T136I) mutation in regulating vancomycin resistance and virulence, there are still several limitations. First, this study only focused on in vitro phenotypic experiments; further in vivo animal infection models are required to verify the regulatory effect of GraS(T136I) on bacterial virulence and drug resistance. Second, the downstream molecular regulatory network mediated by GraS(T136I) has not been fully explored, and more mechanistic experiments will be carried out in our future work.

## Figures and Tables

**Table 1:** Primers used in RT-qPCR.

Primer	Oligonucleotide (5′-3′)
RT-*graS*-F	CGTCAAATCTCAGCGCACAAAG
RT-*graS*-R	TGTTTTCTTTCTTGATTTTTTTCTTGATC
RT-*graR*-F	AATGGGATTTTAATGTTGCTGGTATT
RT-*graR*-R	GATCCATTGGATTATCACGAGATGAT
RT-*dltB*-F	AAGTACATGGTTAGGTGGACATCAGA
RT-*dltB*-R	GTCCAGATGAAATCGTTGGGAAG
RT-*mprF*-F	CTGCACTTTAGTGTCGTGTGTTGAAT
RT-*mprF*-R	CGGTACAAAATAGTACGCAAAACG
RT-*efb*-F	GCACGTCCACAATTTAATAAACCA
RT-*efb*-R	TCAATTCGCTCTTGTAAGACCATT
RT-*hlb*-F	GGTGGGACAAAACTGAAGGTAGC
RT-*hlb*-R	TGCTATCATTATCGAATCCACAACC
RT-*sbi*-F	GGGGAAGCAAAAGCGAGTG
RT-*sbi*-R	TGCACGTTCTGGGTGTTCG
RT-*hld*-F	TTATTTTTTAGTGAATTTGTTCACTGTGTC
RT-*hld*-R	ATGAGTTGTTTAATTTTAAGAATTTTTATCTT
RT-*pta*-F	AAAGCGCCAGGTGCTAAATTAC
RT-*pta*-R	CTGGACCAACTGCATCATATCC

## Data Availability

The original contributions presented in this study are included in the article. Further inquiries can be directed to the corresponding author(s).
